# Interpretation of EBV infection in pan-cancer genome considering viral life cycle: LiEB (Life cycle of Epstein-Barr virus)

**DOI:** 10.1038/s41598-019-39706-0

**Published:** 2019-03-05

**Authors:** Hyojin Song, Yoojoo Lim, Hogune Im, Jeong Mo Bae, Gyeong Hoon Kang, Junhak Ahn, Daehyun Baek, Tae-You Kim, Sung-Soo Yoon, Youngil Koh

**Affiliations:** 1 Cancer Research Institute, Seoul National University College of Medicine, Seoul, Republic of Korea; 2 Center for Medical Innovation, Seoul National University Hospital, Seoul, Republic of Korea; 3 Department of Internal Medicine, Seoul National University Hospital, Seoul, Republic of Korea; 4 Genome Opinion, Ansan, Gyeonggi-do Republic of Korea; 5 Department of Pathology, Seoul National University Hospital, Seoul, Republic of Korea; 6 Center for RNA Research, Institute for Basic Science, Seoul, Republic of Korea; 7 School of Biological Sciences, Seoul National University, Seoul, Republic of Korea

## Abstract

We report a novel transcriptomic analysis workflow called LiEB (Life cycle of Epstein-Barr virus) to characterize distributions of oncogenic virus, Epstein-Barr virus (EBV) infection in human tumors. We analyzed 851 The Cancer Genome Atlas whole-transcriptome sequencing (WTS) data to investigate EBV infection by life cycle information using three-step LiEB workflow: 1) characterize virus infection generally; 2) align transcriptome sequences against a hybrid human-EBV genome, and 3) quantify EBV gene expression. Our results agreed with EBV infection status of public cell line data. Analysis in stomach adenocarcinoma identified EBV-positive cases involving *PIK3CA* mutations and/or *CDKN2A* silencing with biologically more determination, compared to previous reports. In this study, we found that a small number of colorectal adenocarcinoma cases involved with EBV lytic gene expression. Expression of EBV lytic genes was also observed in 3% of external colon cancer cohort upon WTS analysis. Gene set enrichment analysis showed elevated expression of genes related to E2F targeting and interferon-gamma responses in EBV-associated tumors. Finally, we suggest that interpretation of EBV life cycle is essential when analyzing its infection in tumors, and LiEB provides high capability of detecting EBV-positive tumors. Observation of EBV lytic gene expression in a subset of colon cancers warrants further research.

## Introduction

Several types of human cancers involve the infection of oncogenic viruses within the host genome. Data indicate that 10–15% of human cancers are caused by infection with several types of human viruses^1^. Various DNA viruses, such as human papillomavirus, hepatitis B virus, human herpesvirus 8, and Epstein-Barr virus (EBV), and RNA viruses, such as human T-cell lymphotropic virus type 1 and hepatitis C virus, are known to cause cancer in humans^2^. The replication of these DNA and RNA viruses into the host (human) genome via insertional mutagenesis can trigger carcinogenesis due to the effects of virus-encoded elements and host immune deregulation^3^.

EBV (also known as human herpesvirus 4) contributes to the development of human cancers of epithelial cell, mesenchymal cell, and lymphocytic origin. Despite the prevalence of EBV in cancers, the biological impact of EBV lytic gene expression in cancers is not clearly determined yet. We, therefore, aim to identify EBV expression in cancers and understand how EBV expression is related to the biological signature of cancers. Under certain conditions, EBV infection can lead to the development of cancer, and this process is closely related to the life cycle of EBV (i.e., its latent and lytic stages)^4,5^. In fact, EBV viremia is present in 14% of healthy populations^6^. And the infection is asymptomatic and remains latent for a long period, with the virus persisting as episomes in infected B cells^7^ without causing disease^8,9^ in 90% of EBV-infected adults. By contrast, EBV carriers who experience lytic EBV infection frequently develop the infection-related disease, including cancers^10,11^ and autoimmune diseases^12^. The lytic replication cycle begins when the early transcription factors (TFs) are induced; viral promoters activated by the TFs facilitate the formation of the initiation complex, which is composed of six viral gene products: BMRF1, BSLF1, BBLF4, BBLF2/3, BALF5, and BALF2^13,14^. Once EBV-infected cells enter the lytic cycle, lytic antigens are expressed abundantly and trigger cell proliferation^15^.

The advent of next-generation sequencing (NGS) techniques and recent advancements in computational methods have greatly increased understanding of viral metagenomics. Along with shotgun metagenomics techniques, such as nanopore sequencing^16,17^, improvements in the analytic pipeline brought about by whole-genome sequencing (WGS) and WTS have enabled detailed analyses of viral genomes in human samples. For example, Cao *et al*. demonstrated differences in viral infection status between normal and cancerous tissue using *VirusScan*, a novel algorithm that could help further delineate virus-associated carcinogenesis mechanisms^18^. Metagenomics approaches have also broadened knowledge regarding the pathobiology of EBV-related cancers. EBV-associated cancers are known to have a distinct mutational profile compared with EBV-negative cancers^19^. Specifically, The Cancer Genome Atlas (TCGA) Research Group revealed that EBV-positive stomach cancers are enriched with *PIK3CA* mutations, extensive DNA methylation, and programmed-death ligand 1/2 (PD-L1/L2) expression^20^.

In regard to the biological impact of EBV infection in human cancer^19^, entrance into the EBV lytic cycle begins upon differentiation of B lymphocytes towards plasma cells^21^ and often contributes to EBV-associated tumors^22^. In other words, it is imperative to reflect EBV life cycle information when detecting EBV-associated tumors. We, therefore, sought to analyze NGS data from the perspective of the EBV life cycle considering the correlation between the lytic EBV stage and human cancers. Combining current knowledge regarding the genes related to each EBV stage with abundant WTS/WGS data, we analyzed the correlation between EBV lytic genes and the human genome in cancer cases. We hypothesized that in addition to viral infection, the pattern of gene expression related to the viral stage is important in virus-associated carcinogenesis. Hence, in this study, we examined both virus infection status and EBV gene expression pattern using TCGA WTS data. Here, we demonstrate the impact of EBV reactivation characterized by lytic phase gene expression as well as the distribution of EBV infection. From this study, we first established our internal workflow to detect EBV infection and quantify viral gene expression. By using our workflow, we also found out that EBV expression in a small proportion of colon cancers.

## Results

### Detection of EBV infection

**Figure 1:**
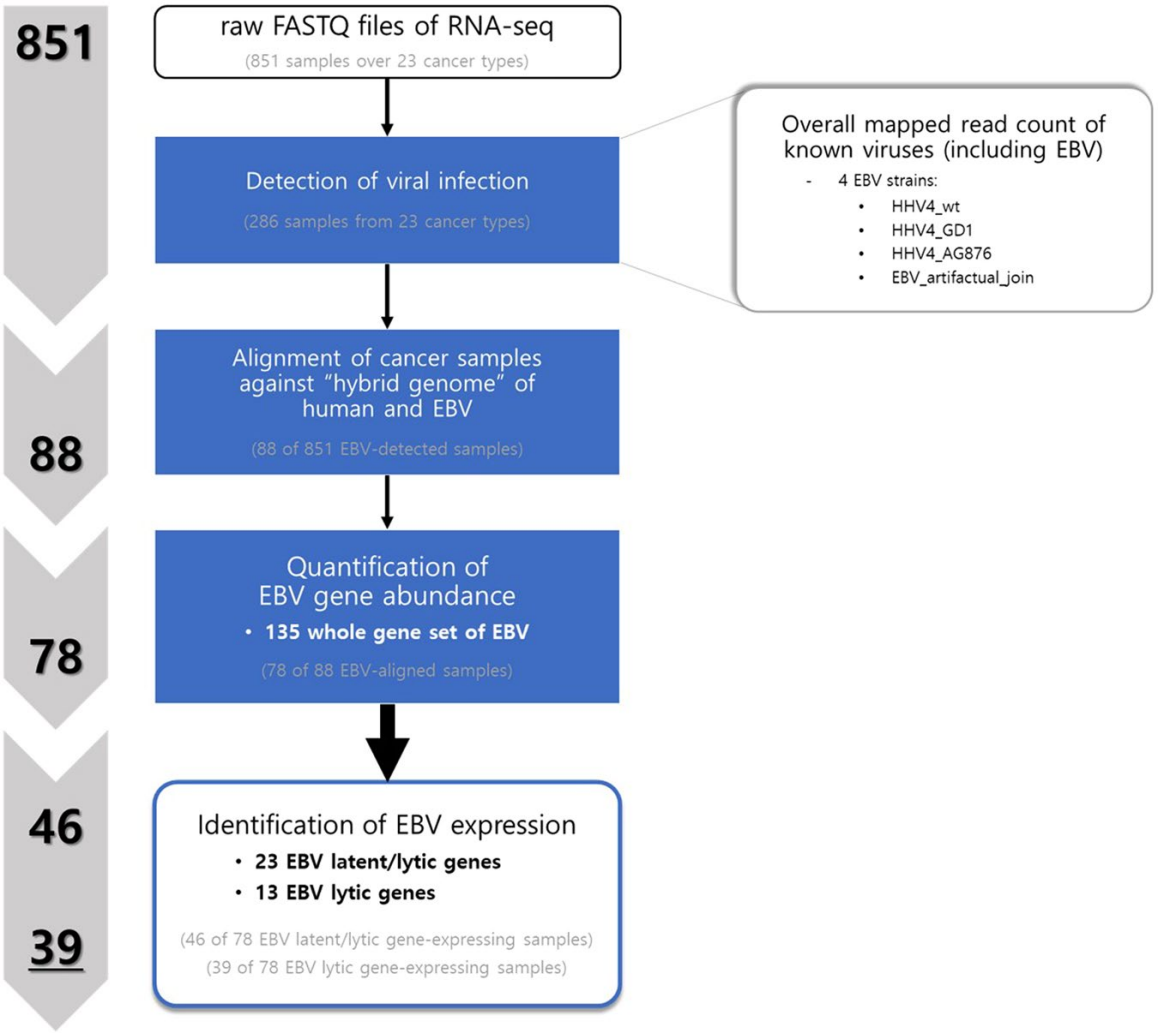
Workflow of the three-step EBV *in silico* detection algorithm. We examined 851 WTS samples covering 23 cancer types from TCGA database. Our workflow (LiEB) involves three steps to detect EBV-positive samples: (1) detection of viral infection; (2) alignment against a hybrid genome; (3) quantification of EBV gene expression.

**Figure 2:**
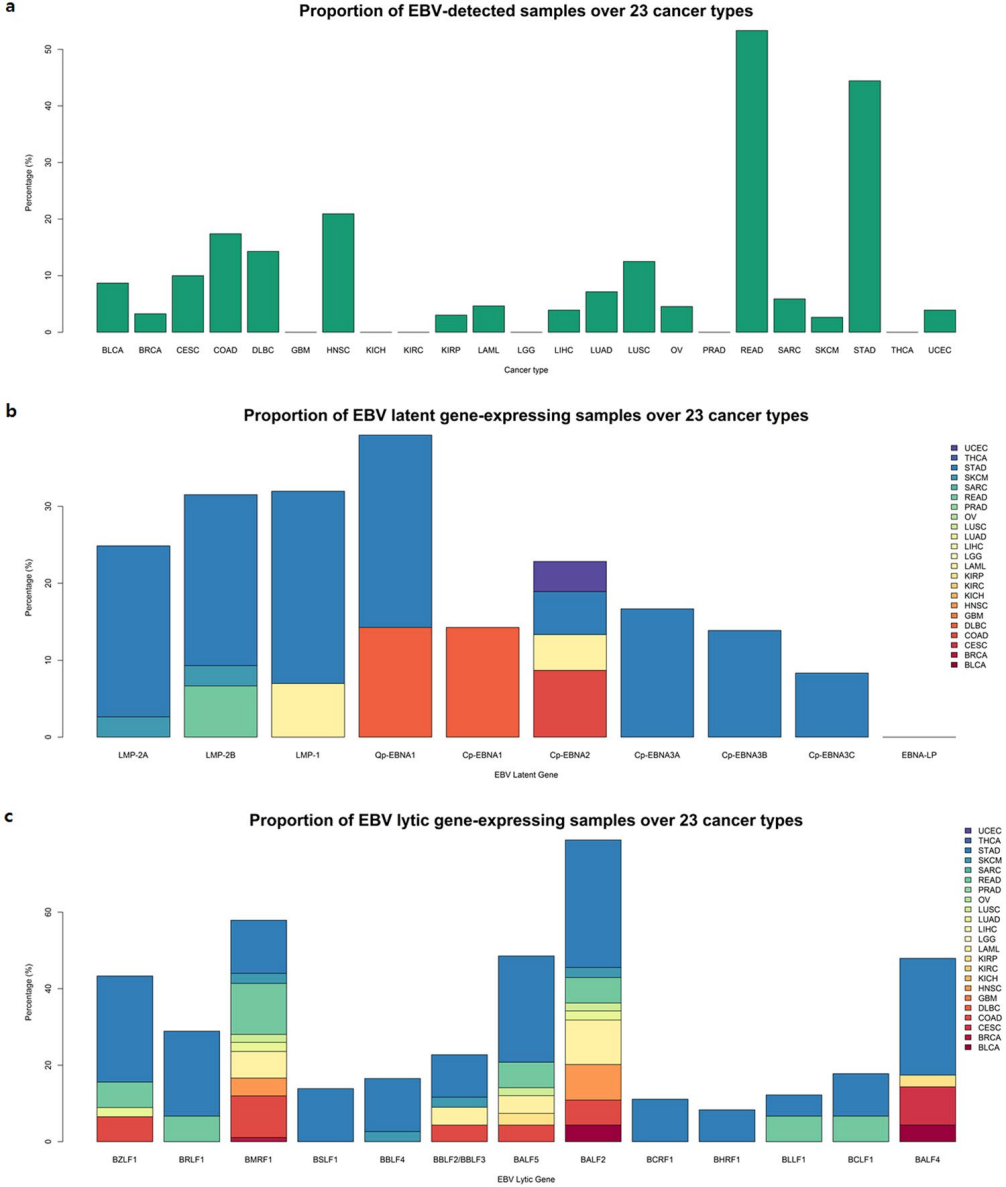
(**a**) Proportion of EBV-positive samples among 23 cancer types examined. Each bar represents the percentage of samples in which one or more mapped read was detected for each of the four EBV strains (HHV4_wt, HHV4_GD1, HHV4_AG876, and EBV_artifactual_join) examined. Note that we did not include READ in the further analysis set due to an insufficient number of sample sets (N = 7), although this cancer type shows a high proportion of EBV infection in this figure. (**b,c**) Proportion of samples expressing EBV latent and lytic genes among 23 cancer types examined. Each stacked bar indicates the percentage of samples of each cancer type expressing EBV latent (**b**) and lytic (**c**) genes. Colors represent each TCGA cancer project.

We investigated 851 TCGA WTS samples involving 23 cancer types to detect EBV infection by employing our three-step LiEB workflow (Fig. 1). We first identified the 286 infection-positive samples (33.61%) using VirusSeq (Fig. 2a). Then these EBV-positive samples identified using VirusSeq were mapped against the four EBV strains (GenBank accession: AJ507799, AY961628, AG876, DQ279927, and M80517M75989) using STAR approach; 88 WTS samples (10.34%) were then distinguished to selectively contain sequences aligned against the hybrid transcriptome. As a next step, we employed the RSEM algorithm, and RSEM analysis indicated expression of EBV latent and lytic genes in 78 samples. Since these samples are identified to have any expression of the whole set of 135 EBV genes, we selected 46 of 78 samples by sorting out according to the expression of the 23 gene products (13 lytic and 10 latent genes) (Supplementary Table S2). Finally, we could classify 46 EBV-positive samples, among which 39 were sub-classified as expressing EBV lytic genes.

### Quantification of EBV-related gene expression

Examining the STAR-aligned reads against the EBV genome, we quantified the expression of both EBV latent (Fig. 2b) and lytic (Fig. 2c) genes by calculating transcript abundance; prior to the identifying the expression of EBV genes related to its life cycle, we quantified the overall expression of EBV genes belong to its viral genome (Supplementary Fig. S3). As a well-known EBV-related cancer^23,24^, the proportion of EBV-expressing samples in STAD was high (47X%, 17/36). Unexpectedly, we found a proportion of EBV lytic gene expressing cases in colorectal cancers (39%, 24/61): colon adenocarcinoma (COAD) and rectal adenocarcinoma (READ) (Fig. 2c). In summary, we could find EBV lytic gene expressing cases both in upper and lower gastrointestinal cancers (COAD, READ, and STAD).

In specific, three EBV lytic genes were mainly expressed in the colorectal cancer samples: 1) *BZLF1*, encoding an early transcription factor; 2) *BMRF1*, encoding a DNA polymerase processivity factor^25^, and 3) *BALF2*, encoding an EBV single-stranded DNA-binding protein^15^. Since these three lytic genes were dominantly expressed in STAD, we adduce that enrichment of EBV lytic genes in COAD is convincing.

### *In silico* validation using cell line WTS data

We applied our three-step LiEB approach in cell line WTS data (Supplementary Fig. S4a and b) to validate our algorithm. As a result, we could clearly dichotomize cell lines regarding EBV status. EBV-positive cell lines (MP-1, Raji, and Akata) exhibited high expression of both lytic and latent genes, such as *BHRF1*, *BHLF1*, *Cp-EBNA2*, and *LMP*-1, whereas two EBV-negative cell line samples (both are HCT-116 strains) were not mapped against the hybrid genome (Supplementary Fig. S4), indicating no expression of EBV genes (Supplementary Fig. S4b). Validation result using cell line WTS data supports the robustness and accuracy of detecting EBV of our LiEB workflow.

### Accuracy of LiEB method for EBV infection detection in STAD samples

We classified the STAD samples into two groups based on the presence of EBV infection by applying LiEB. We compared our results with previous data reported by TCGA Networking group^20^ and by Ding’s group^18^. As mutational hallmarks of EBV related stomach cancer are well described^20,26^, we analyzed the integrated WXS MAF file to correlate salient STAD-related mutation signatures with EBV status. We deemed hallmarks of EBV-related stomach cancer^20^ as mutations in *PIK3CA*, *ARID1A* and *BCOR* genes, PD-L1/2 overexpression, and CDKN2A silencing.

When we compared our result with the previous report by TCGA Networking group^20^, we could identify five more samples (N = 13) with EBV infection than TCGA report (N = 8) which implies the sensitivity of detecting EBV lytic expression was raised by 61% with LiEB (concordance rate 86%). In these five additional samples with EBV positivity, hallmarks of EBV infection – either *PIK3CA* or *ARID1A* mutation – was observed in three cases: STAD_21, STAD_19, and STAD_17 (Table 1). In fact, these five additional samples were also detected as EBV positive in recent Li Ding group’s report^18^.

**Table 1:** Significant mutations related to STAD (TCGA)*.

STAD_id	EBV (TCGA)	EBV_Lytic (Hsong)	EBV (Li Ding)	TPM (CDKN2A)	CDKN2A	PIK3CA	ARID1A	BCOR	RHOA	TPM (PDL1)	TPM (PDL2)
STAD_13	1	1	1	3.97	0	Missense	Nonsense	Splice_Site	0	309.51	14
STAD_28	1	1	1	4.59	Missense	Missense	fsDEL, ifDEL, Nonsense	0	0	125.1	9.86
STAD_24	1	1	1	18.99	0	Missense	3′UTR	0	Missense	29.19	2.84
STAD_11	1	1	1	26.12	Missense	Missense	fsDEL	0	0	19.25	2.71
STAD_34	1	1	1	33.4	0	0	ifDEL	0	0	7.58	3.28
STAD_27	1	1	1	6.48	0	Missense	Nonsense	0	0	3.74	3.06
STAD_31	1	1	1	0.55	0		fsDEL	0	0	1	0.19
STAD_8	1	1	1	2.23	0		0	0	0	6.37	3.96
STAD_21	0	1	1	6.25	0	Missense	fsDEL, Splice_Region	Missense	0	4.06	2.74
STAD_19	0	1	1	7.83	0		Missense, ifDEL	Missense		4.78	1.28
STAD_17	0	1	1	1.45	0	Missense	ifDEL		0	3.74	1.37
STAD_3	0	1	1	17.4	0	0	0	0	0	2.94	2.8
STAD_25	0	1	1	13.73	0		0	0	0	0.95	0.57
STAD_10	0	0	1	669.8	fsDEL, ifDEL	0	ifDEL	0	0	3.81	1.59
STAD_1	0	0	1	349.74	Missense	0	0	Missense	0	6.1	1.25
STAD_32	0	0	0	124.75	0	0	ifDEL	0	0	2.14	2.19
STAD_4	0	0	0	12	0	0	0	0	0	3.65	2.09
STAD_5	0	0	0	12.49		Missense	Missense, fsDEL, ifDEL	Missense	0	2.82	2.19
STAD_22	0	0	0	2.89	0	0	ifDEL	0	0	3.59	0.38
STAD_16	0	0	0	1.09	0	0	fsINS	0	Missense	1.21	0.25
STAD_2	0	0	0	3.61	0	Missense	fsDEL, ifINS		0	2.03	1.21
STAD_6	0	0	0	41.64	0	0	0	0	0	3.14	2.47
STAD_7	0	0	0	44.47	0	Missense	Missense, ifDEL	0	0	2.8	2.67
STAD_9	0	0	0	26.12	0	0	ifDEL	0	0	1.48	1.4
STAD_33	0	0	0	183.79	0	0	ifDEL	0	0	5.51	0.99
STAD_14	0	0	0	19.54	0	fsINS, 3′UTR, Splice_Site, Splice_Region	fsDEL, ifDEL	0		3.13	1.31
STAD_18	0	0	0	160.75	Missense	0	fsDEL, ifDEL	Missense	0	2.83	2.27
STAD_36	0	0	0	207.21			Missense, fsDEL, 3′UTR			11.24	3.48
STAD_20	0	0	0	41.25	0	0	3′UTR, ifDEL	0	0	0.58	0.26
STAD_23	0	0	0	29.9	0	0	ifDEL	0	0	0.78	1.11
STAD_15	0	0	0	234.54	Missense		0	0	0	0.39	0.35
STAD_35	0	0	0	42.19	0	0	0	0	0	1.54	1.04
STAD_30	0	0	0	11.78	0	0	ifDEL	0	0	0.17	0.41
STAD_29	0	0	0	11.96	0	0	0	0	0	0.19	0.41
STAD_26	0	0	0	2.36	0	0	ifDEL	0	0	0.83	0.41
STAD_12	0	0	0	2.99	0	0	Missense	Nonsense	0	3.36	1.41

*fsINS, Frame_Shift_Ins; fsDEL, Frame_Shift_Del; ifDEL, In_Frame_Del; ifINS, In_Frame_Ins.

When we compared our results with Li Ding group’s report, we could observe high concordance between the two algorithms (94%). However, there was a discrepancy in two samples between LiEB and Li Ding’s algorithm. Two samples (STAD_10 and STAD_1) detected EBV-positive in Li Ding group’s workflow^18^ seemed not to harbor EBV lytic-genes by LiEB workflow. Interestingly, these two samples showed strikingly high expression in CDKN2A (669.8 and 349.74 respectively calculated in TPM, Table 1) which is not compatible with characteristics of EBV associated STAD. In fact, the EBV positive samples by LiEB workflow showed low CDKN2A expression values between 0.55 and 33.4 (in TPM). As described earlier, CDKN2A silencing including gene downregulation^27^ is a hallmark of EBV associated STAD and this analysis suggests that STAD_10 and STAD_1 may not be EBV associated STAD’s at least biologically. In summary, our LiEB workflow detects EBV lytic-positive samples more accurately than the previously reported workflows.

### External validation of EBV infection in colon cancer cases

**Figure 3:**
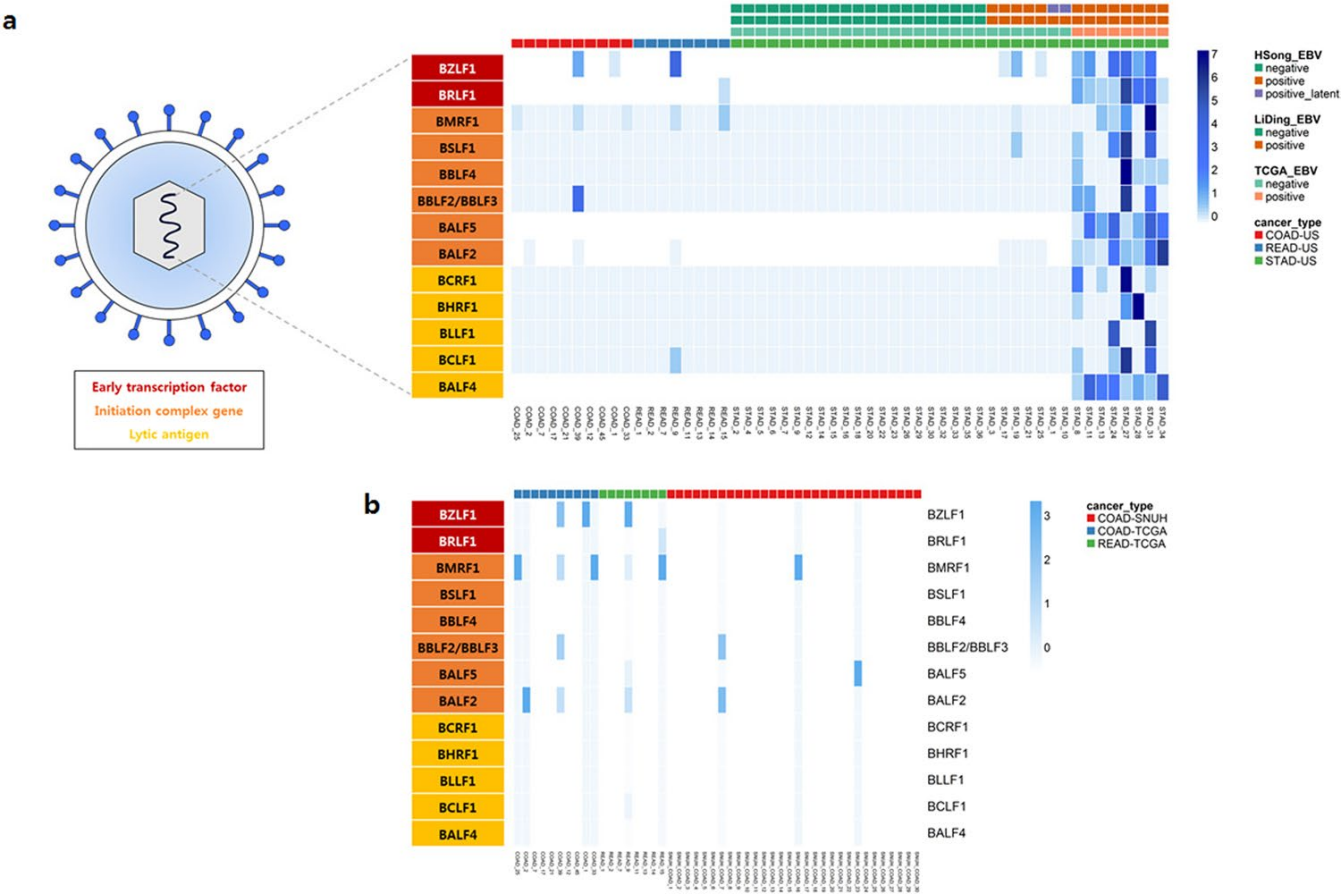
(**a**) EBV lytic gene expression and its partial genomic structure. (Left) Thirteen EBV lytic genes were classified into three categories according to the sequential processes of the lytic stage: early transcription factors, initiation complex genes, and lytic antigens (c.f., the list of lytic genes refers to Draborg, A. H. *et al*. *Clinical and Developmental Immunology* [2012]). (Right) Heatmap illustrating the expression of EBV lytic genes in three types of gastrointestinal cancer types: colon adenocarcinoma (COAD, red), rectal adenocarcinoma (READ, blue), and stomach adenocarcinoma (STAD, green). The gradation of blue color indicates the row-scaled expression value of each EBV gene. Three annotation bars on STAD samples indicate those that were EBV positive as detected by our LiEB workflow and in published preliminary reports: TCGA Network group^20^ and Li Ding group^18^. (**b**) Comparison of EBV lytic gene expression in TCGA COAD, READ, and SNUH_COAD. Heatmap illustrating the expression of EBV lytic genes in three gastrointestinal cancer cohorts: SNUH colon cancer (COAD-SNUH, red), TCGA colon adenocarcinoma (COAD-TCGA, blue), and rectal adenocarcinoma (READ-TCGA, green). The gradation of blue color indicates the column-scaled expression value of each EBV gene.

To validate EBV lytic gene expression in colorectal cancer, we analyzed separate 30 colon cancer transcriptome sequencing data collected from our institution (SNUH cohort). In SNUH cohort, seven cases showed expression of EBV genes, with one case (SNUH_COAD_7) exhibiting comparatively high expression of EBV lytic genes (3% positivity by LiEB) (Fig. 3). The results of PCR analyses using the EBNA1 probe correlated well with EBV gene expression in transcriptome sequencing data (EBV viral load of case SNUH_COAD_7 was 27,872 copies/mL).

### Gene Set Enrichment Analysis (GSEA)

**Figure 4:**
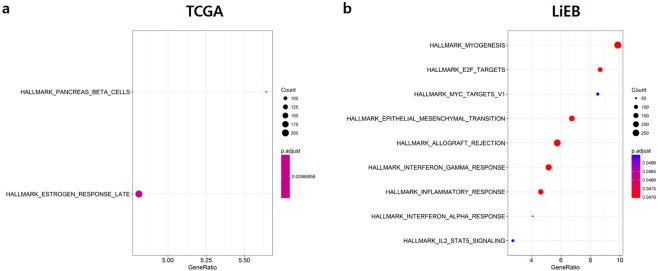
Dot plot of enriched pathways determined from GSEA results. Each dot plot demonstrates enriched pathways in TCGA (5a) and LiEB (5b) comparison of GSEA results. The size of the dot represents gene count, and the color represents the adjusted p-value.

In TCGA STAD project, GSEA of EBV-positive samples detected using our LiEB workflow demonstrated enrichment in inflammation-related gene sets: Interferon-gamma response, Interferon-alpha response, inflammatory response, and Interleukin2-STAT5 signaling. This is in contrast to the previous analysis: GSEA results based on the EBV detection by TCGA Networking group showed that genes in the Pancreas-beta cells and Estrogen response late gene sets were enriched in EBV-positive samples (Fig. 4a). We could easily conclude that GSEA result based on LiEB is more biologically relevant than previous analysis (Fig. 4b). In addition, it should be noted that E2F related gene expression was enriched in EBV positive cancers when analyzed by LiEB. E2F is a factor that is crucial for cancer development in virus-related oncogenesis^28^.

## Discussion

Viral infections in humans can lead to carcinogenesis resulting from dysfunction in the host immune system. And EBV is closely related to human cancers originating from epithelial cells, lymphocytes, and mesenchymal cells^19,20^. According to the “Hit and Hide” theory suggesting that EBV evades the host immune system by remaining dormant in cells, the lytic infection can trigger the induction and maintenance of EBV-positive cancers^29,30^. In addition, the expression of EBV lytic gene products can induce the production of growth factors and oncogenic cytokines^31^ that in turn contribute to carcinogenesis. Hence, in the analysis of EBV-related cancers, the life cycle of EBV including lytic gene expression should be considered. This is the starting point where we developed LiEB algorithm. In this study, infection of EBV genomic sequences was identified in ~10% of cancers mostly originating from epithelial cells and only half of these cases (~4.6%) involve EBV lytic gene expression.

We could confirm the accuracy of LiEB in various aspects. First is through mutation profiling of TCGA STAD dataset. We observed a stronger association between *PIK3CA* mutation and CKNA2A silencing with EBV infection than the previous reports. It is well known that both of *PIK3CA* mutation and down-regulation of *CDKN2A* is a hallmark of EBV associated tumor^1^. Second, based on GSEA results, we confirmed that our LiEB workflow detects more biologically meaningful EBV-related tumors as the EBV-positive sets were heavily enriched with gene set related to inflammation and E2F targeting. Inflammation is a central feature of virus-associated cancers. When cells are infected, inflammatory signaling is activated and inflammatory cytokines recruit various types of immune cells (e.g., eosinophils, monocytes, mast cells, and T cells) that target infectious viral antigens^32^. We could only observe the enrichment of inflammatory pathways from GSEA when we classified EBV status using LiEB, and not by using TCGA Network analysis. Also, E2F is a transcription factor that regulates carcinogenesis in case of virus-related cancers^28,33^.

We employed a three-step viral infection detection algorithm in LiEB. What was an unexpected finding in our study is that we found a small proportion of EBV-positive samples in colorectal cancers. Two TCGA samples (involving colon and rectal adenocarcinoma) exhibited expression of EBV lytic genes (*BZLF1*, *BMRF1*, and *BALF2*), analogous to that observed in EBV-positive stomach cancer samples. We identified additional EBV-positive colon cancer in SNUH cohort, which supports that EBV may play a role in a small number of colon cancers. Moreover, viral load analysis result using quantitative PCR was concordant with the expression of EBV genes detected using LiEB. (Supplementary Table S2). However, we could not observe a definite signal of EBV infection when tested by EBV *in situ* hybridization (ISH). EBER ISH results in seven EBV-gene expressing (either lytic- or latent-positive) cases showed a few stained tumor-infiltrating lymphocytes between tumor glands (data not shown). It should be noted however that, ISH is an inferior method to NGS in detecting EBV infection^34^. EBER-ISH is capable of detecting EBV positivity when tumors contain a certain amount of EBV-aligned reads.

Furthermore, we tried to look into noncoding RNAs called miRNAs related to EBV life cycle. Both cellular and viral miRNAs regulate gene expression of either host or virus itself and affect regulatory networks as a part of the carcinogenic mechanisms^35^. In particular, cellular miRNAs interact with viral oncoproteins and this biological processes influence often enhances the survival of virus-infected cells^36,37^. Analysis of miRNA expression in EBV-positive cell lines (AKBM, C666-1, SNU-719, and Jijoye) revealed that the expression of miRNAs associated with EBV lytic cycle varied in terms of latency phases (from latency I to III) (Supplementary Data S5). Because there were only latently infected EBV-positive cell lines available for public use, we could indirectly speculate how those miRNAs may function in the EBV-infected cells. We identified the expression of viral miRNAs significantly associated with EBV reactivation such as miR-BART2, miR-BART18, and miR-BART20^10,38^, and observed that expression of those viral miRNAs was gradually increased upon latency phases from Latency I to III. The increased expression of the viral miRNAs in latently-infected cells implicates that the viral latency is established and maintained, not contributing to inducing lytic cycle^39^. In specific, miR-BART20 directly targets lytic switch proteins (Zta and Rta), and its expression blocks lytic induction^40^. Along with the expression of viral miRNAs, we also identified expression of cellular miRNA genes involved in EBV reactivation such as miR-155 and miR-200 family members (miR-200b and miR-429); especially, miR-200 family members are involved in modulating EBV lytic reactivation by downregulating ZEB1 and ZEB2 on viral lytic gene product, Zta (also known as BZLF1)^41,42^. Since we observed lower expression of these cellular miRNAs in both Latency I and III, this suggests that the cells expressing miR-200 family members may control whether to turn the lytic switch on. From our analysis, hence, we support that the expression of those miRNAs, especially ebv-miR-BART20 and hsa-miR-200 family, either suppress the induction of lytic cycle^43^ or is depleted in latently infected B cells^42^.

In summary, we investigated WTS data in pan-cancer samples to identify cases involving infection with EBV. Two major conclusions could be drawn based on the results of this study. First, our LiEB workflow detecting the expression of EBV lytic genes provides an indication of biologically important EBV infection in humans, beyond simple viral infection. The ability to determine whether the host has entered the lytic or latent stage of the EBV life cycle would be significant for preventing severe symptoms or development of cancer by providing personalized therapies in advance. Second, we elucidated the EBV gene expression pattern in a small portion of colon cancers, and these patterns were analogous to that in stomach cancers.

Conclusively, our findings provide information that brings us closer to a comprehensive understanding of EBV infection, especially in cooperation to the EBV lytic stage and EBV-associated carcinogenesis. Furthermore, this transcriptomic investigation determining EBV latent and lytic stage might suggest novel clues to understand the biological roles of EBV lytic expression in gastrointestinal carcinomas.

## Materials and Methods

### Input data collection

We used tumor TCGA WTS data for 851 samples covering 23 cancer types (Supplementary Table S1). These samples were generated from 827 donors, with the remaining samples derived from the same donor with acute myeloid leukemia (AML) and stomach adenocarcinoma (STAD). The data were downloaded in raw FASTQ file format, which was appropriate for initiating specialized alignment against the sequence of a newly generated hybrid human/EBV genome.

### Use of WTS data to detect EBV infection

WTS data was analyzed by using our internally developed workflow called LiEB (Life cycle of Epstein-Barr virus), detecting infection and expression of Epstein-Barr virus in terms of its lytic/latent life cycle.

#### Rapid detection of viral infection

VirusSeq was used for rapid detection of viral infection into the human genome^44^. The VirusSeq algorithm utilizes viral genomic sequences currently available on Genome Information Broker for Viruses (http://gib-v.genes.nig.ac.jp/) database^45^. Rough infection of EBV into the human genome was estimated using genomic information for four EBV strains (codes in parentheses are GenBank accession): human herpesvirus 4, complete wild-type genome (AJ507799); human herpesvirus 4, strain GD1 (AY961628); human herpesvirus 4, strain AG876 (DQ279927), and EBV artifactual join (M80517M75989).

#### Alignment against a human/EBV hybrid reference genome

Spliced Transcripts Alignment to a Reference (STAR)^46^ was performed against a human (GRCh38 assembly)/EBV (type 1 EBV strain) hybrid reference genome. Because the genomic sequences of typical EBV type 1 strains contain a site for the splitting of the circular genome adjacent to the terminal repeats (TRs), it can be challenging to detect *LMP2* transcripts located in the genome in close proximity to the TRs^47^; *LMP2* encodes *LMP2A* and *LMP2B*. However, the inverted genomic sequence of Akata cells (an EBV-positive cell line established from a Japanese patient with Burkitt’s lymphoma) contains a breakpoint between the *BBRF3* and *BGLF3* genes instead of near the TRs, making this sequence more suitable for detecting *LMP2* transcripts^48,49^. We, therefore, used an “inverted” FASTA format of the Akata genome sequence to investigate the infection of EBV genomic sequences. Using the inverted Akata genome as an EBV reference genome enabled us to overcome the difficulties associated with both detecting the mapped reads against *LMP2* transcripts in the alignment procedure and capturing *LMP2* mRNA expression for gene expression quantification^50^.

#### Analysis of EBV-related gene expression

We used the identical reference genome of the EBV type 1 strain (chrEBV_Akata_inverted) to quantify the expression of EBV latent and lytic genes by applying RNA-Seq by Expectation-Maximization (RSEM) algorithm v1.3.0^51^, calculated as transcripts per million (TPM) values. In order to determine the EBV life cycle stage in each tumor examined, we used a list of 135 EBV gene products^52^ and calculated the expression in TPM unit for each. In order to more clearly delineate life cycle stage, we examined a set comprised of 23 EBV gene products^15^ (Supplementary Table S2) involved in both lytic and latent infection stages. The resulting data were used to determine whether each EBV-positive sample had entered the lytic stage for viral reactivation or was in the latent stage and therefore dormant.

### *In silico* validation using cell line WTS data

Raw FASTQ files of cell line WTS data were downloaded from the NCBI SRA open source (SRA study accession: SRP079984 and SRP107862). Data for known EBV-positive lymphoblastoid cell lines (MP-1, Raji, and Akata) and an EBV-negative colorectal cell line (HCT-116) were used for preliminary external validation of LiEB workflow (see Supplementary Fig. S4a and b).

### External validation of EBV infection in different colon cancer samples

WTS data collected from 30 cases of colon cancer diagnosed at the Seoul National University Hospital (SNUH) were used for external validation (IRB No. 1809-046-971). The infection and expression of EBV-related genes were examined according to the LiEB workflow described in both 2) Alignment against a human/EBV hybrid reference genome and Analysis of EBV-related gene expression section above. Polymerase chain reaction (PCR) was then performed to validate the expression of EBV genes in the colon cancer cohort. The PCR probe was designed to target Epstein-Barr nuclear antigen 1 (EBNA1; a nuclear protein expressed in both the latent and lytic stages of EBV infection^53^) and used to determine the number of EBV copies in each sample (i.e., the EBV viral load). We further performed conventional *in situ* hybridization (ISH) method by using the probe for EBV-encoded small RNA (EBER), to define EBV infection status.

### Mutation signature profiling

The integrated mutation annotated format (MAF) file for whole-exome sequencing (WXS) in the STAD project was download from the Genomic Data Commons data portal, administered by the National Cancer Institute; the protected version of the TCGA MAF file is available for use by authorized members. In order to verify the correlation between the expression of genes associated with EBV-positive STAD (*PDL1*, *PDL2*, and cyclin-dependent kinase inhibitor 2A (*CDKN2A)*) and EBV lytic-positive samples, the TPM values of these genes were matched to the STAD samples.

### Gene set enrichment analysis (GSEA)

To demonstrate the effectiveness of our LiEB workflow, we compared the EBV-positive results from a previous TCGA report^20^ with the workflow using GSEA. Samples were first segregated as either EBV-positive or EBV-negative. Next, we analyzed a set of differentially expressed genes and used the output as the GSEA input data (GSEABase R package). As the input gene set for enrichment analysis, we used a gene matrix transposed file of hallmark gene sets available from the Molecular Signatures Database collection website, supported by the Broad Institute (http://software.broadinstitute.org/gsea/msigdb/collections.jsp). After GSEA, we utilized the DOSE R package to construct a list of enriched gene sets in the comparison group. In this step, two types of GSEA have performed: comparisons between 1) TCGA-positive samples and the remaining samples, and 2) our LiEB-positive samples and the remaining samples.

## Supplementary information

Supplementary Information

Dataset 1

## Data Availability

The main datasets used in this study are available from the corresponding authors for reasonable academic purposes. Besides, the datasets used for validation are available on the NCBI SRA and study accessions are mentioned in the text where it is used.
