# Supplementary material for: Interpretation of EBV infection in pan-cancer genome considering viral life cycle: LiEB (Life cycle of Epstein-Barr virus)
Source: Sci Rep. 2019 Mar 5;9:3465. doi: 10.1038/s41598-019-39706-0 (PMC6401378; doi:10.1038/s41598-019-39706-0)
Supplement: Supplementary file 1 — Supplementary Information [file 41598_2019_39706_MOESM1_ESM.pdf]

Interpretation of EBV infection in pan-cancer genome considering viral life cycle: LiEB (Life cycle of Epstein-Barr virus)

Hyojin Song[1,2], Yoojoo Lim[3], Hogune Im[4], Hyung-Lae Kim[5], Jeong Mo Bae[6], Gyeong Hoon Kang[6], Junhak Ahn[7,8], Daehyun Baek[7,8], Tae-You Kim[3], Sung-Soo Yoon[1,3*], Youngil Koh[2,3*]

[1]Cancer Research Institute, Seoul National University College of Medicine, Seoul, Republic of Korea

[2]Center for Medical Innovation, Seoul National University Hospital, Seoul, Republic of Korea

[3]Department of Internal Medicine, Seoul National University Hospital, Seoul, Republic of Korea

[4]Genome Opinion, Ansan, Gyeonggi-do, Republic of Korea

[5]Ewha Medical Research Institute, Ewha Womans University College of Medicine, Seoul, Republic of Korea

[6]Department of Pathology, Seoul National University Hospital, Republic of Korea

[7]Center for RNA Research, Institute for Basic Science, Seoul, Republic of Korea

[8]School of Biological Sciences, Seoul National University, Seoul, Republic of Korea

*Correspondence to go01@snu.ac.kr or ssysmc@snu.ac.kr.

Supplementary Information

Supplementary Table S1. Sample set obtained from 827 donors from TCGA database. Table shows information for each sample, including TCGA study project name/code, sample_ID, donor_ID, analysis_ID, and md5sum value.

Supplementary Table S2. Gene sets associated with each stage of the EBV life cycle. Table lists 13 EBV lytic genes and 10 latent genes: EarlyTransFac, early transcription factors; OriLyt, viral gene products constituting the initiation complex at lytic origin of replication; LyticAntigen, lytic antigens expressed during the lytic cycle; LatentAntigen, latent antigens expressed during latent cycle (EA/D, diffused early antigen; VCA, viral capsid antigen).

Supplementary Figure S3. Proportion of samples expressing EBV genes among 23 cancer types examined. Each stacked bar shows the percentage of samples expressing genes from among a set of 135 EBV genes. Colors represent each TCGA cancer project.

Supplementary Figure S4. EBV gene expression in cell lines. Each column represents a cell line sample (EBV-positive cell lines: MP-1, Raji, and Akata_IRF8-dep; EBV-negative cell line: HCT-116). Each row represents genes from among a set of 135 EBV genes (4a) and a set of 13 EBV lytic genes (4b).

Supplementary Table S5. Table of both human and EBV miRNA gene expression. This table shows expression of miRNAs in EBV-associated cell lines (EBV-positive cell lines: AKBM, C666-1, SNU-719 and Jijoye; EBV-negative cell line: HK-1). Both viral and cellular miRNAs were selected based on the previous reports (mentioned in the main text) as significantly associated with EBV lytic

reactivation; (viral miRNAs: ebv-miR-BART2, ebv-miR-BART18, and ebv-miR-BART20; cellular miRNAs: hsa-miR-155, hsa-miR-200b, and hsa-miR-429).

Supplementary Table S1.

See Supplementary Dataset (Excel file).

Supplementary Table S2.

Related EBV life cycle stage	Functional annotation	Gene ID	EBV polypeptide type
Lytic	EarlyTransFac	BZLF1	
Lytic	EarlyTransFac	BRLF1	
Lytic	OriLyt	BMRF1	EA/D
Lytic	OriLyt	BSLF1	
Lytic	OriLyt	BBLF4	
Lytic	OriLyt	BBLF2/BBLF3	
Lytic	OriLyt	BALF5	
Lytic	OriLyt	BALF2	
Lytic	LyticAntigen	BCRF1	
Lytic	LyticAntigen	BHRF1	
Lytic	LyticAntigen	BLLF1	gp350
Lytic	LyticAntigen	BCLF1	p160VCA
Lytic	LyticAntigen	BALF4	gp110VCA
Latent	LatentAntigen	LMP-2A	
Latent	LatentAntigen	LMP-2B	
Latent	LatentAntigen	LMP-1	
Latent	LatentAntigen	Qp-EBNA1	
Latent	LatentAntigen	Cp-EBNA1	
Latent	LatentAntigen	Cp-EBNA2	
Latent	LatentAntigen	Cp-EBNA3A	
Latent	LatentAntigen	Cp-EBNA3B	
Latent	LatentAntigen	Cp-EBNA3C	
Latent	LatentAntigen	EBNA-LP	

Supplementary Figure S3.

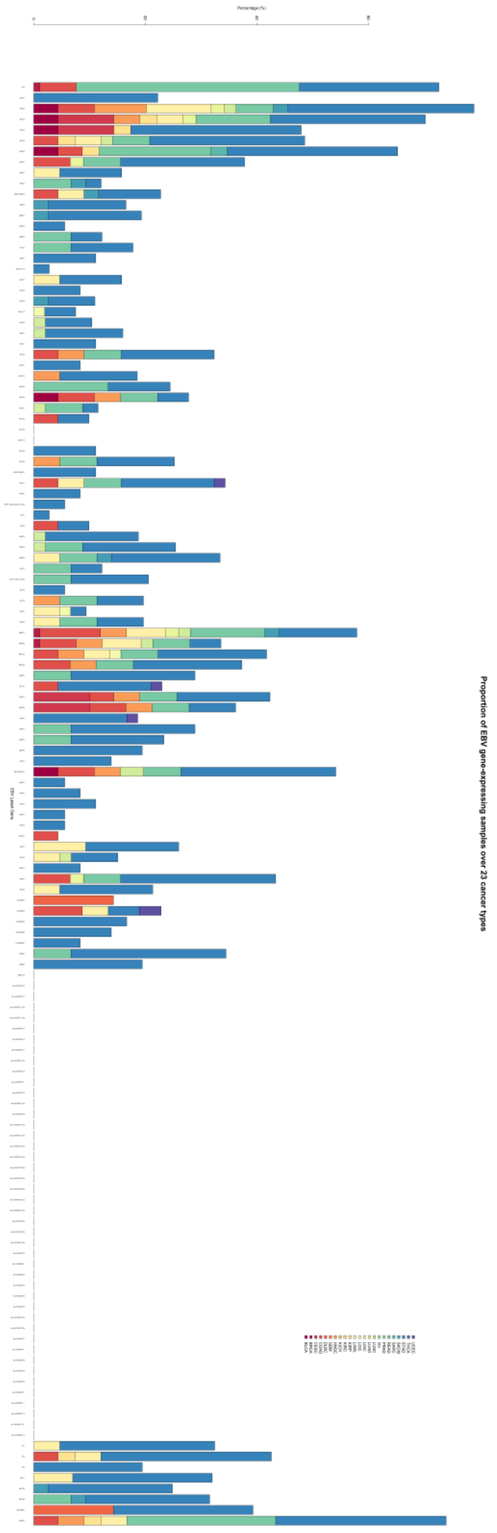

Supplementary Figure S4a.

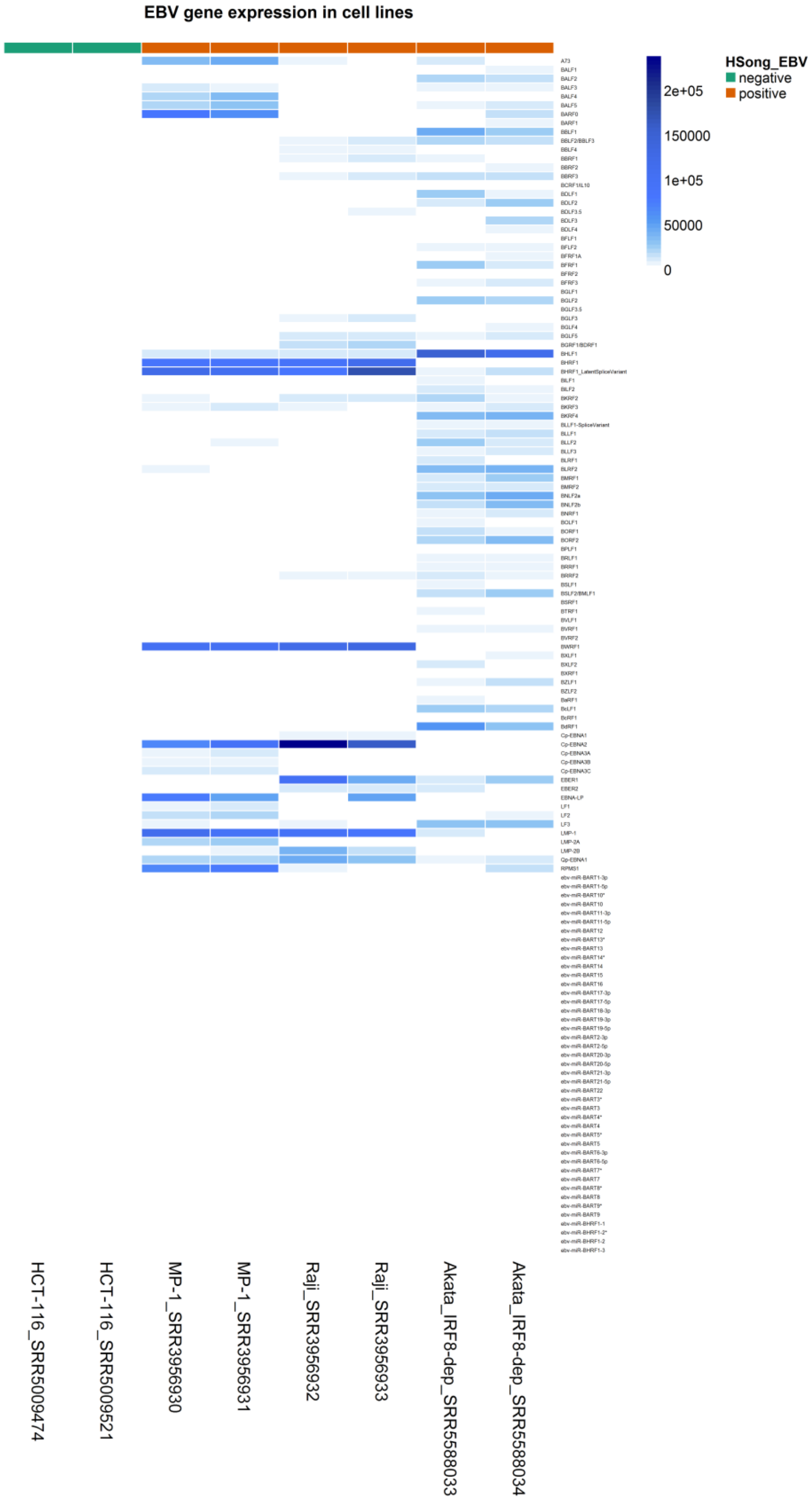

Supplementary Figure S4b.

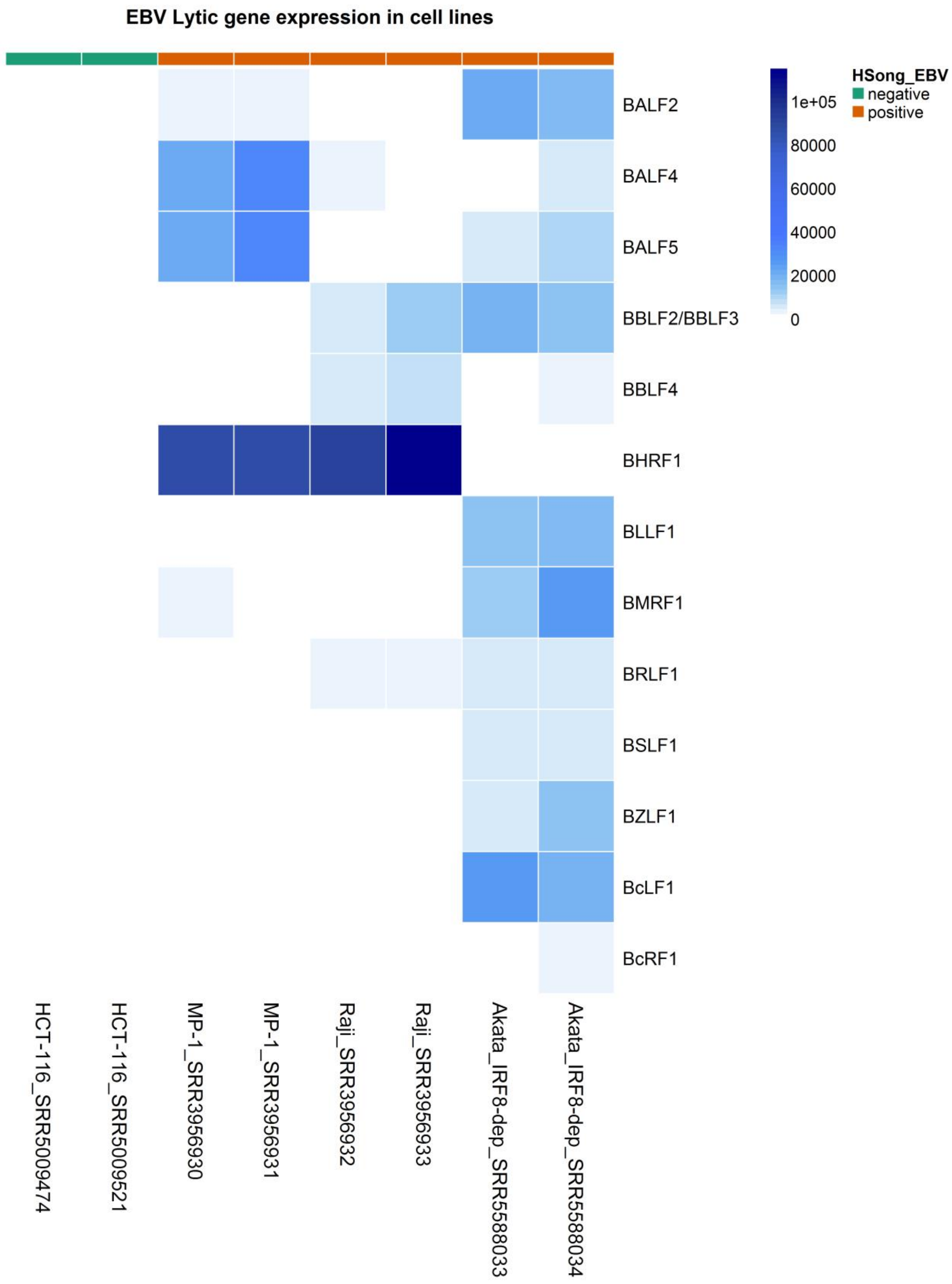

Supplementary Table S5.

		EBV-negative	EBV-positive			
		SRR2913245	SRR2913244	SRR2913241	SRR2913242	SRR2913243
**Li et al. (*Int J biol Sci* 2016)**	**cellular miRNAs**	**HK-1 (empty-vector)**	**AKBM**	**C666-1**	**SNU-719**	**Jijoye**
**viral**	**ebv-miR-BART2-5p**	0	51.18507054	330.8066795	201.0306561	943.4723982
**viral**	**ebv-miR-BART18-3p**	0	9.34972718	36.22896259	64.43024835	113.9310098
**viral**	**ebv-miR-BART20-5p**	0	0.190946302	7.690575448	2.354587541	13.88407345
**cellular**	**hsa-miR-155-5p**	11.03066376	1.743422756	1.339801715	1.151884552	5883.392872
**cellular**	**hsa-miR-200b-3p**	160.3335511	1.095865732	208.0649413	859.0658035	2.402473185
**cellular**	**hsa-miR-429**	124.1745634	0.224154354	80.69468817	289.1035458	0.259090245

Supplementary Method

Analysis of EBV-associated gene expression at miRNA-level

We further obtained 5 samples of small RNA sequencing data for EBV-positive cell lines (SNU-719, C666-1, AKBM and Jijoye) and EBV-negative cell line (HK1) available on the National Center for Biotechnology Information (NCBI) Sequence Read Archive (SRA) open source (SRA study accession: SRP066099). We quantified the expression of miRNA genes (in human and EBV genome, respectively), using in-house small RNA sequencing analysis pipeline mentioned as follows.

Low base quality reads were trimmed using in-house software and 3' and 5' Illumina adapters were removed by Cutadapt v1.2.148 with the following parameters: –m 17 –match-read-wildcards –O 10 –e 0.1. Then, we filtered out sequencing artifacts using Fastx_artifacts_filter from FASTX-Toolkit v0.0.13.2 (http://hannonlab.cshl.edu/fastx_toolkit/) with –Q 33 parameter; the reads aligned to either human tRNA or rRNA were discarded. For the alignment, bowtie2 v2.1.049 was used with the following parameters: –k 101 –very sensitive and default options. The processed reads were aligned to both human (hg38) and EBV genome (NC_007605) with the following parameters: –k 101 –score-min C,-8,0 –mp 8,8 –np 8 –very-sensitive and default options, allowing up to one mismatch or one insertion/deletion. We used the human and EBV miRNA annotation files available from miRBase (release22), respectively. Expression level for each mature miRNA was calculated in reads per million (RPM) unit; {the number of aligned reads to the mature miRNA} / {the number of aligned reads to hairpin miRNAs x 106} (see Supplementary Table S5).
